# Human visual exploration reduces uncertainty about the sensed world

**DOI:** 10.1371/journal.pone.0190429

**Published:** 2018-01-05

**Authors:** M. Berk Mirza, Rick A. Adams, Christoph Mathys, Karl J. Friston

**Affiliations:** 1 Wellcome Trust Centre for Neuroimaging, Institute of Neurology, University College London, London, United Kingdom; 2 Institute of Cognitive Neuroscience, University College London, London, United Kingdom; 3 Division of Psychiatry, University College London, London, United Kingdom; 4 Scuola Internazionale Superiore di Studi Avanzati (SISSA), Trieste, Italy; 5 Translational Neuromodeling Unit (TNU), Institute for Biomedical Engineering, University of Zurich and ETH Zurich, Zurich, Switzerland; 6 Max Planck UCL Centre for Computational Psychiatry and Ageing Research, London, United Kingdom; Technische Universitat Dresden, GERMANY

## Abstract

In previous papers, we introduced a normative scheme for scene construction and epistemic (visual) searches based upon active inference. This scheme provides a principled account of how people decide where to look, when categorising a visual scene based on its contents. In this paper, we use active inference to explain the visual searches of normal human subjects; enabling us to answer some key questions about visual foraging and salience attribution. First, we asked whether there is any evidence for ‘epistemic foraging’; i.e. exploration that resolves uncertainty about a scene. In brief, we used Bayesian model comparison to compare Markov decision process (MDP) models of scan-paths that did–and did not–contain the epistemic, uncertainty-resolving imperatives for action selection. In the course of this model comparison, we discovered that it was necessary to include non-epistemic (heuristic) policies to explain observed behaviour (e.g., a reading-like strategy that involved scanning from left to right). Despite this use of heuristic policies, model comparison showed that there is substantial evidence for epistemic foraging in the visual exploration of even simple scenes. Second, we compared MDP models that did–and did not–allow for changes in prior expectations over successive blocks of the visual search paradigm. We found that implicit prior beliefs about the speed and accuracy of visual searches changed systematically with experience. Finally, we characterised intersubject variability in terms of subject-specific prior beliefs. Specifically, we used canonical correlation analysis to see if there were any mixtures of prior expectations that could predict between-subject differences in performance; thereby establishing a quantitative link between different behavioural phenotypes and Bayesian belief updating. We demonstrated that better scene categorisation performance is consistently associated with lower reliance on heuristics; i.e., a greater use of a generative model of the scene to direct its exploration.

## Introduction

This paper is about salience attribution in visual searches. In other words, how do we identify salient targets during saccadic (visual) searches of our visual scenes–and what sorts of policies and prior beliefs underwrite this attribution and subsequent epistemic foraging. To address this question, we applied a recently described model of active visual search (i.e., inference) to explain the eye movements of normal subjects in terms of optimal epistemic sampling. In this paper, we consider the evidence that normal subjects conform to normative (i.e., Bayesian) principles and how this can be used to characterise individual differences. Ultimately, we will translate this paradigm into clinical research, with a special focus on aberrant salience attribution in people with schizophrenia.

Visual exploration entails seeking relevant information, given a context. But what is information? Shannon’s definition of information [1] implies that an outcome that is less predictable contains more information. Shannon entropy is the average or expected information. Shannon entropy is highest when all outcomes are equally likely; i.e., when the outcome is most unpredictable. However, Itti and Baldi [2] demonstrated that whilst human visual attention is attracted to areas of high Shannon information, it is attracted most strongly to areas that cause the greatest shifts in our beliefs about the world. This notion is formalised as ‘Bayesian surprise’ [2]: the KL divergence between prior and posterior beliefs about how our sensory data are generated. In the active inference framework, stimuli that are expected to produce greater Bayesian surprise have more epistemic value and are therefore more likely to be sampled through active vision.

The active inference framework explains both exploratory and subsequent goal-fulfilling behaviour in terms of avoiding surprise and reducing uncertainty. Mathematically this is described by minimizing expected free energy; where variational free energy is a proxy for the negative log evidence or surprise. This variational principle generates behaviour that avoids surprising outcomes (i.e. that do not conform to prior beliefs). In brief, agents seek out states that resolve the greatest uncertainty about the environment (i.e., maximising Bayesian surprise), while avoiding states that do not conform to its prior beliefs (i.e., avoiding surprise *per se*). This involves equipping the agent with the beliefs that she will minimize the expected free energy of future outcomes; i.e. the free energy of beliefs about the future. To evaluate expected free energy, beliefs about the state of the world are projected into the future to predict the most likely outcomes. This enables the agent to select free energy minimising, epistemic, goal-directed policies (i.e. sequences of actions) that resolve the most uncertainty about its environment and fulfil its goals.

The expected free energy comprises two terms; namely, epistemic (i.e., intrinsic) and pragmatic (i.e., extrinsic value). Epistemic value speaks to the resolution of uncertainty about the hidden states of the world, and can be interpreted as the value of knowing one’s environment [3]. This component of expected free energy is essentially the Bayesian surprise expected under a particular action [2]. In other words, in the active inference framework, *salience* is defined in terms of the epistemic value or affordance of sampling the world in a particular way. In contrast, pragmatic value is the expected utility of future outcomes, where utility is simply the logarithm of prior preferences that specify preferred outcomes or goals. The minimization of variational free energy maximizes both epistemic and pragmatic value, and thus resolves the exploration and exploitation dilemma within a single imperative. On this view, active inference first involves resolving uncertainty about the world by selecting actions or policies that are epistemically valuable. After uncertainty has been resolved there is no further epistemic value or affordance–and exploitative behaviour emerges that is driven by prior preferences or pragmatic value.

In our previous work [4]; we introduced an active inference scheme for visual searches using a scene construction task. We showed how a scene can be explored optimally, when a synthetic subject engages in epistemic foraging. In this work, we ask whether human subjects perform the same task in an epistemic fashion; i.e., resolving uncertainty about the hidden states of the world. To answer this question we fitted active inference models to the behaviour of subjects, using models that did–and did not–contain epistemic value. This enabled us to evaluate the evidence for epistemic foraging using Bayesian model comparison. We then asked whether we can disambiguate behavioural phenotypes in terms of their prior preferences or beliefs, using canonical correlation analysis.

This paper comprises four sections. The Materials and Methods section comprises three subsections. In the first, we describe the active inference scheme used in this paper. We briefly rehearse the generic form of the Markov decision process (MDP) for active inference and reiterate the scene construction task. In the second, we describe the analyses of the saccadic scan-paths of subjects performing the scene construction task. In the third we describe the empirical methods for the gaze-contingent protocol we used in the eye-tracking study and the subjects that performed the task. The Results section contains the behavioural and model based analyses of the subjects’ scan paths. In the model based analyses we use our Bayesian model of visual searches to estimate the subjects’ prior beliefs. We then report the canonical correlations between subjects’ prior beliefs and their behavioural measures to understand overt behaviour in terms of characteristic subject ‘types’. In the Discussion section, we discuss our results in terms of active inference and their implications for computational phenotyping of individual subjects.

## Materials and methods

### Active inference and visual search

In this section, we briefly rehearse the Markov decision processes model used to simulate active inference. Here, this model is used to simulate visual searches; however, the underlying formalism is the same as used in previous work to model a wide range of behavioural and physiological responses [3,5,6].

**Fig 1:**
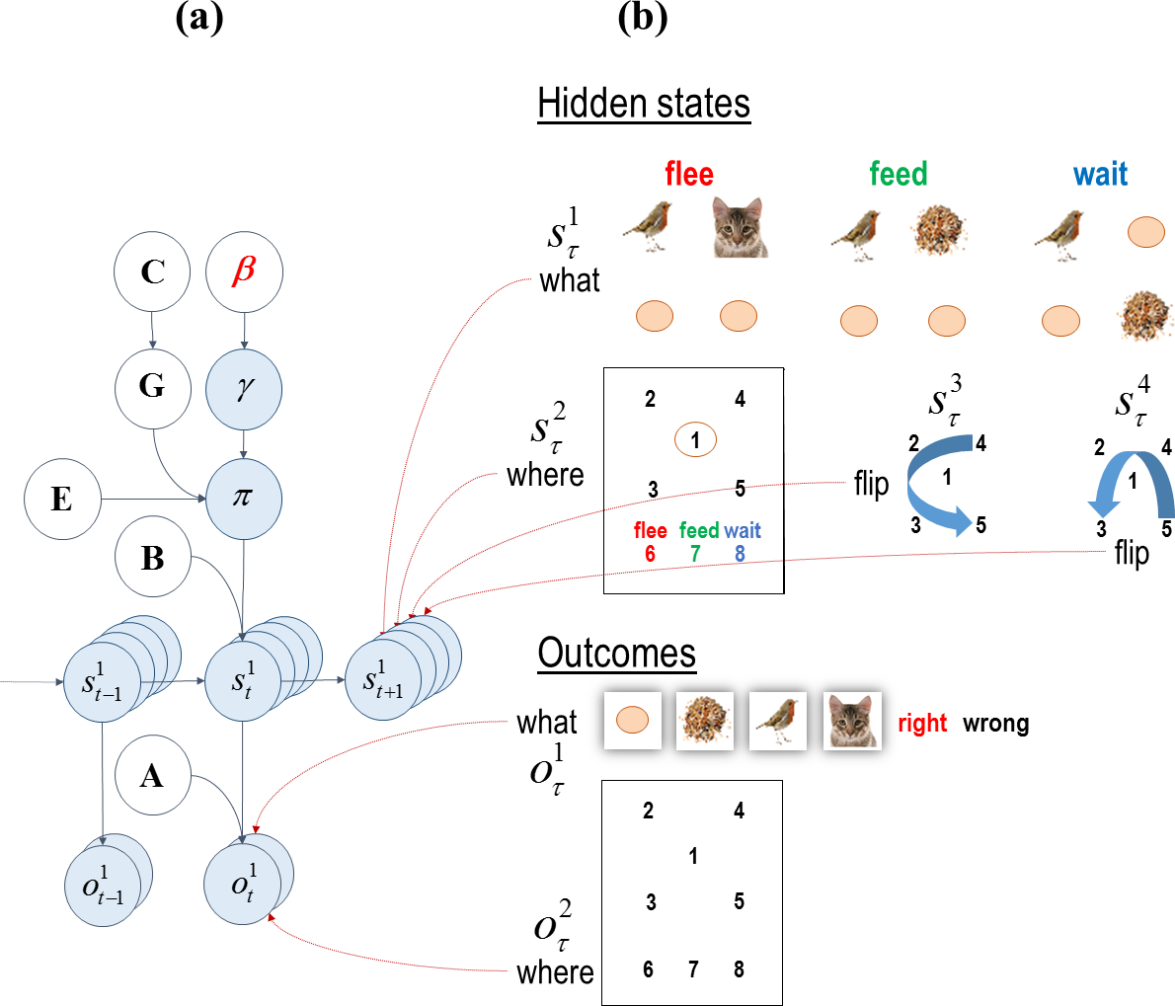
Graphical model corresponding to the generative model. **(a)** The left panel shows that the structure of an environment is defined through the transition (**B**) and likelihood (**A**) matrices. The transition matrices encode the transition probabilities between hidden states, whereas the likelihood matrix encodes how likely outcomes are given the hidden states. The **C** matrix encodes the agent’s prior preferences about the outcomes. β determines the confidence or precision placed in policy selection–based upon expected free energy in the future. The smaller this term, the more precise policy selection becomes. **G** is the expected free energy and comprises extrinsic (pragmatic) and epistemic (uncertainty resolving) components. Beliefs about policies **π** are a function of β, **G** and **E** where **E** prescribes the prior probabilities over the policies (including fixed-form or heuristic policies). Hidden states *s* depend on action and the hidden states in the previous time step. Finally outcomes *o* depend on the hidden states. **(b)** The right panels show different hidden state and outcome modalities in the scene construction task. There are four hidden states, namely, the scene context, sampling location, horizontal and vertical spatial transformations (*what*, *where*, and *transformations* respectively). The two outcome modalities report *what* object is seen *where* in the scene. Reprinted from [4] under a CC BY license, with permission from Mirza, Adams, Mathys and Friston, original copyright 2016. This figure is not identical to the original figure.

In the current task, the subject’s goal is to categorize a two by two grid scene as *Flee*, *Feed* or *Wait*. The relative locations of the objects in the scene define the category; e.g., in *Feed* scenes a *seed* is next to a *bird*. The objects in the scene are masked initially and are subsequently revealed by looking at the quadrants. These objects are sampled, one at a time, in a sequential manner to gather evidence for allowable scene categories. The locations of the objects are determined by states that are ‘hidden’ from the subject–and have to be inferred on the basis of sensory evidence. The first hidden state (*what*) determines the category of the scene by defining the relative locations of the objects. The second hidden state is *where*; namely, the sampling quadrant. The last two hidden states are *spatial transformations* that define the absolute locations of the objects. These hidden states swap or flip the locations of the objects horizontally and vertically with respect to the base scenes. The base scenes are shown in Fig 1B, using the *what* dimension of hidden states. Under a vertical transformation or flip of the flee scene, the bird and cat would exchange locations. Once the agent accumulates sufficient evidence for one of the categories, it samples one of the choice locations to receive feedback about its decision; where the feedback can be either *right* or *wrong*. The agent is given prior preferences that it *a priori* expects to be *right* and not *wrong*.

Given a generative model of how outcomes are generated by hidden states of the world, active inference models behaviour in terms of planning as inference [4,7,8]. In brief, policies or sequences of actions are inferred by treating each policy as a potential model of the future and using Bayesian model selection to select the most likely policy. The most likely policy is the policy that one expects to provide the greatest model evidence or least expected free energy. The expected free energy can be decomposed into epistemic and pragmatic parts that resolve uncertainty about hidden states of the world and realise prior preferences respectively. This policy selection involves inferring the hidden states of the world under each allowable policy using variational message passing. Variational Bayes assumes statistical independence among marginal distributions over different sets of hidden states. This allows the posterior distribution over one set to be inferred (from observed outcomes), while the posterior distributions over the others are held constant. This is repeated for all sets of hidden states, until convergence. This sort of approximate Bayesian inference uses a *mean field approximation*, which assumes a factorisation and conditional independence among the posterior distributions over different dimensions or factors of hidden states (here, *what*, *where and spatial transformations*). We consider two sorts of outcomes; namely, the exteroceptive visual outcome or *what* is observed and the proprioceptive outcomes; i.e., *where* the agent is currently looking.

Fig 1A shows the generic form of the MDP model and the conditional dependencies in the generative model. This panel shows how the outcomes are generated from the hidden states in terms of probabilistic transitions that depend on policies. There is only one difference in the directed acyclic graph in this paper and in our previous work [4]; namely, prior (fixed-form) beliefs over policies (denoted by **E**). To accommodate the fact that subjects may have preferred heuristic strategies for visual search, our model of their policies included a fixed-form (i.e., heuristic) policy that was estimated on a subject by subject basis. This fixed-form policy corresponds, technically, to a state-action policy. In other words, it is a policy that prescribes (in a deterministic way) the next target location given the current location. This can be encoded as a single policy in terms of a probability transition matrix among different saccade locations. The heuristic policy was estimated using the empirical transition frequencies for every sequence of saccadic eye movements analysed–and the most frequently used heuristic policy was included in the repertoire of policies for each subject. Although this policy is subject-specific, it plays exactly the same role in every instance; namely, a state-action policy that has no uncertainty reducing or epistemic aspects. This can be seen easily because the most probable next action or saccade does not depend upon posterior beliefs–that would otherwise contextualise an epistemic saccade. The remaining eight (epistemic) policies correspond to single saccades that take the agent to one of the eight locations in the scene. Generally, policies comprise a sequence of actions or moves; however, in this paper we only consider simple (one step ahead) policies that correspond to the next action. An epistemic policy is defined as a policy (based on current beliefs about hidden states) that resolves the greatest amount of uncertainty about hidden states (i.e., maximises information gain, Bayesian surprise or salience). In contrast to heuristic policies, action is not predetermined by the hidden state one finds oneself in, but by the action’s epistemic affordance. This means epistemic policies can therefore accumulate information more efficiently, because they depend upon current beliefs.

Operationally, we parameterised the propensity to engage in a fixed-form (heuristic) policy with a single log probability (an **E**_**h**_ coefficient, where h stands for heuristic). This value was specified relative to a value of zero for the remaining eight policies that could be deployed in an epistemic fashion, i.e. **E** = [0,…,0,**E**_**h**_]. Including the heuristic policy allowed us to evaluate how likely different subjects were to engage in epistemic versus non-epistemic searches–and whether this propensity changed with exposure to the task.

**Fig 2:**
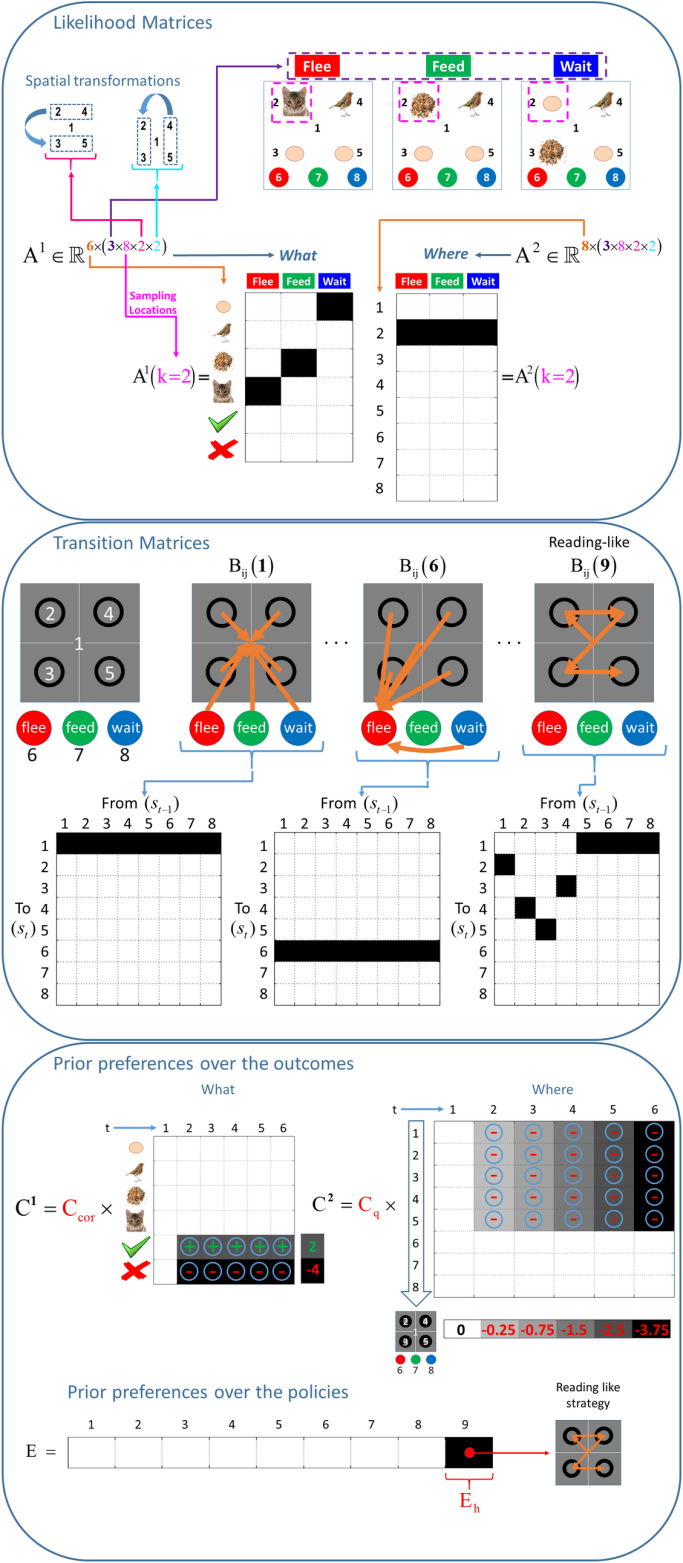
ABCDE of generative model. The upper panel shows the likelihood matrices for the top left quadrant (location 2), under the vertical (but not horizontal) spatial transformation. The likelihood matrices encoded the probability of outcomes given (the four dimensions of) hidden states. The middle panel shows the action-dependent state transition matrices. In this scene construction task, only the sampling location (where) hidden state is action-dependent. The other transition matrices associated with the context and spatial transformations are identity matrices. The action-dependent transition matrix maps to the sampling locations associated with each action (for the first eight policies). However, this mapping changes in the case of a fixed-form policy, which corresponds to a repeating the ninth action in the MDP model. The mapping between sampling locations for the reading-like policy is shown on the rightmost side of the middle panel. The lower panel shows two priors. Firstly, the prior preferences over the what and where outcome modalities in the first six saccades are shown. Here, the columns of the matrices show the preferences (or utilities) over successive time steps; whereas the rows designate the outcomes (six cues under the what modality and eight locations under the where modality). The utilities in the first time step are zero (shown with the white colour), under both modalities; since the sampled location in the first time step is the central fixation. Different shades of grey indicate the absolute value (intensity) of the utilities, where the darker shades are associated with higher utilities. The prior preference matrix under the what modality equips the agent with beliefs that it expects to categorize correctly. The increasing utility over the columns in the prior preference for the where modality means that the tardy sampling (i.e., being undecided) becomes costly. Plus and minus signs indicate the valence of the utilities. These utilities specify the imperatives or instructional set of the empirical game (described below); in terms of incentives or prior preferences. For example, considering the prior preference matrix under the what modality, a correct categorisation is implicitly rewarded with two points and an incorrect categorisation would be penalised with four points. The prior preferences over policies are shown at the bottom. One can define a propensity for a policy in the vector **E**, which encodes the prior preferences over the policies. An example **E** is shown at the bottom. The utility of the ninth policy (heuristic strategy, **E**_**h**_) is defined as log(2), relative to a value of zero for the remaining (eight) policies. The first eight policies correspond to the policies that take the agent to the locations associated with the central fixation, four quadrants and three choice locations. This renders the ninth policy ≈ 3 times more likely.

Interestingly, we found that the fixed-form policies in several subjects resembled a reading-like strategy, while in others there was a tendency to proceed clockwise around the quadrants. See the rightmost side of the middle panel of Fig 2 for an exemplar empirical probability transition matrix encoding subject-specific policies (in this case the reading-like strategy). In our MDP scheme heuristic policies are sufficient for scene exploration; however, they do not support a categorisation response. In other words, once the category of the scene is disclosed through exploration, the heuristic strategies are redundant. To include the propensity of a subject to engage in a fixed-form (heuristic) policy in the MDP scheme, the priors are added to the variational free energy scoring the evidence for each policy based upon past outcomes (**F**) and expected free energy in the future (**G**) weighted by their inverse precision (β). This leads to a posterior belief over policies:

π=σ(E−F−G/β) (1)

where **E** = ln E and E corresponds to the prior preferences over the policies. See our previous work [6] for the details. All the remaining update equations remained the same as in our previous work [4].

Fig 1B shows the hidden state and the outcome space. There are two outcome modalities. Under the *what* modality there are six outcome cues, namely, *null*, *bird*, *seed*, *cat*, *right* and *wrong* feedback. Under the *where* modality, there are eight outcomes; namely, central fixation, four peripheral locations and three *choice* locations. The hidden states have four dimensions; namely, *what* (*Flee*, *Feed* and *Wait*), sampling locations (one of eight locations in the scene), and two spatial transformations (*vertical* and *horizontal*). The scene always contains a *bird*. Whether the scene contains a *cat* or a *bird* depends on *what* scene is currently generating sensory samples. In *Flee* scenes, the *cat* is next to the *bird*; in the *Feed* scenes, the *seed* is next to the *bird* and finally in the *Wait* scenes the *seed* is on the same diagonal as the *bird*.

In this visual search paradigm, the exteroceptive and proprioceptive outcomes are generated in the following way. The context hidden states determine the relative locations (e.g., *seed* next to *bird*) of the objects, whereas the spatial transformation hidden states determine the final locations of the objects by flipping them vertically, horizontally, neither or both. The sampled location and the visual object in that location are conveyed to the subject once an action is sampled from the actions that constitute a policy.

The generative model used to generate stimuli in our experimental paradigm (see next section) is illustrated in Fig 2. The likelihood matrices mapping from hidden states to outcomes are shown in the upper panel. For illustrative purposes, the likelihood matrices are provided for the sampling of the second location (the top left quadrant) under a vertical transformation of the scene. The middle panel shows the action-dependent transition matrices that generate transitions among hidden states following each action. Crucially, the first eight action-dependent transition matrices–that encode the transition probabilities between sampled locations–map deterministically onto the same location as the action; whereas the transition matrix for the ninth action corresponds to a fixed-form (heuristic) state-action policy. This prescribes the next location given the current location in a deterministic way. The transition matrices for the other hidden state dimensions; namely, the *what* or scene context and spatial transformations are identity matrices (because these do not change within each trial). The rules of the game, in terms of scoring points, have been modelled in terms of the prior preferences over outcomes in the **C** matrices. These rules are explained in detail in the next section. As noted above, prior preferences over the policies correspond to **E**. Finally, the generative model assumes uniform beliefs about the hidden states of the world, apart from the initial sampling location; namely, central fixation.

### Characterising empirical behaviour in terms of active inference

In terms of data analysis, we adopted the following strategy. First, the hundred trials within each of five blocks were summarised in terms of scan-paths or sequences of saccadic locations. Using the stimuli or cues that were disclosed during each epoch of every trial, we were then able to optimise the parameters of an MDP scheme that best explained each subject's behaviour. The (free) parameters of the MDP scheme included a hyperprior on the (inverse) precision of beliefs about policies (**B**eta), prior preferences for outcomes (**C**ost) and a prior expectation (**E**xpectations) or bias towards non-epistemic (heuristic) policies. Note that these (**B**, **C**, **E**) parameters encode prior beliefs (about behaviour, preferences and prior policies respectively). This enabled us to optimise the model of each subject’s responses, while accommodating subject-specific preferences.

**Fig 3:**
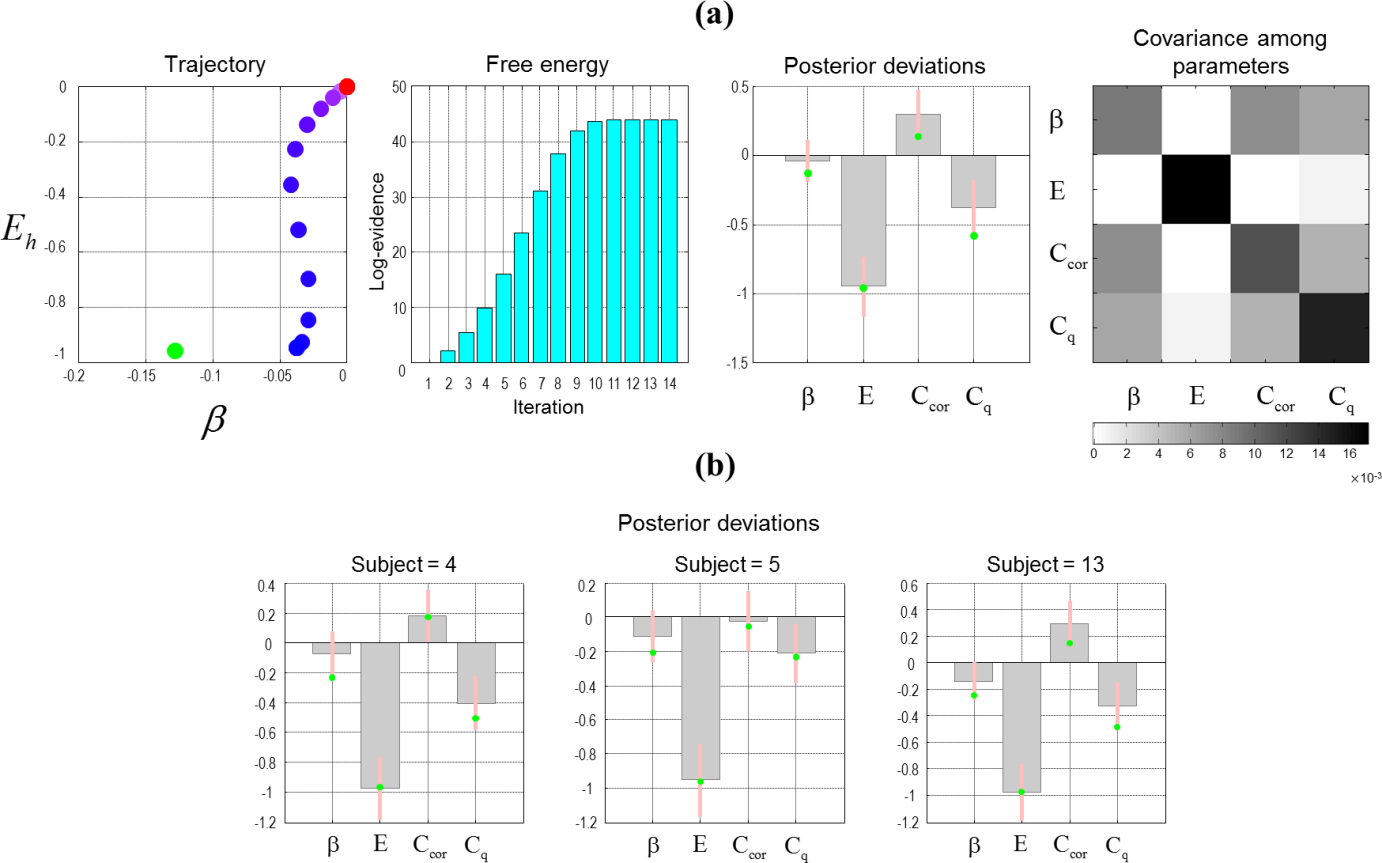
Parameter estimation, simulation and re-estimation. **(a)** This simulation shows that the estimates of the model parameters, given the saccadic scan-paths, can be recovered accurately. The estimated parameters are prior inverse precision, heuristic bias and prior preferences–**β**, **E**_**h**_, **C**_**cor**_ and **C**_**q**_. These parameters are estimated in log-space. Here, a sequence of saccadic choices was simulated using the estimated **β** and **E**_**h**_ parameters from fitting a model to the saccadic choices of the first subject on the third testing block (shown with green dots). The left panel shows how the estimated **β** and **E**_**h**_ parameters change with each iteration, when a model is fit to the simulated data (from red to blue). The middle-left panel shows how the free energy changes with each iteration during the model inversion. The middle-right panel shows the means in log-space (grey bars) and the variances (pink bars) of the probability distributions over the estimated parameters. The right panel shows the posterior covariance matrix among the free parameters (estimated parameters) **(b)** These panels show equivalent posterior expectations and 90% credible intervals based upon simulated saccade sequences using the parameters estimated from three subjects (on different testing blocks) chosen at random. These results suggest that model inversion can recover plausible values.

As a prelude to analysis of empirical data, we first ensured that fitting the MDP model to observed behaviour has face validity. To do this, we estimated the model parameters using the saccadic choices of the first subject (on the third testing block) and used them to simulate a block of 100 trials. Using the simulated data, we then estimated the MDP parameters to ensure that we could recover the same values used to generate the data. The results of an exemplar analysis are shown in Fig 3A. Here, one can see that the scheme was able to recover the parameters used to generate the data, with a reasonable degree of confidence (the grey bars correspond to the posterior expectations in log-space and the pink bars correspond to 90% Bayesian credible intervals).

This was the inversion scheme that was applied to the empirical data to address the hypotheses about whether subjects evidence epistemic behaviour–and whether this behaviour increases (or decreases) with exposure to our paradigm. Our subsequent analysis of the empirical behavioural data comprised three components.

- First, we assessed the evidence that subjects engaged in epistemic searches–as described by minimising expected surprise (i.e. free energy), under ideal Bayesian assumptions. We therefore compared models of each subject’s responses (during the last blocks of each session), under models that did–and did not–contain a salience or uncertainty-reducing term (i.e., epistemic or intrinsic value). Removing this epistemic value from expected free energy reduces it to an expected utility, scored in terms of prior preferences or cost [3]. The evidence for epistemic imperatives in visual searches was assessed for each subject individually in terms of the difference in log evidence for the two models–and then pooled (i.e. summed) to provide inference based on all the subjects’ data.
- Second, we asked which parameter combinations account for the exploratory behaviour the best by evaluating the model evidence obtained under each model by using Bayesian model reduction and averaging [9], where each parameter combination corresponds to a model. This was assessed for each subject individually and then the model evidence under all models was pooled together over all subjects to produce an overall Bayes factor to find which model best accounts for subjects’ behaviour overall.
- Finally, to characterise intersubject variability, we used canonical correlation analysis (CVA) to see whether there were significant relationships between the behaviour of our subjects and their prior beliefs, as estimated in terms of the parameters of the MDP model. This involved creating a matrix of independent or explanatory variables corresponding to the free parameters for each subject and trying to explain the corresponding dependent or response variables based upon subjects’ performance. In this instance, we summarised behaviour in terms of their accumulated score over all trials and performance improvement from the first to the last block. These behavioural measures were supplemented with (partially redundant) performance measures; reflecting the percentage correct categorisations and the number of saccades emitted on average over trials. This analysis returned significant pairs of canonical vectors and variates describing how prior parameters or beliefs are manifest behaviourally. Note that these performance scores are distinct from the scan-path data used to estimate the prior beliefs of each subject.

The number of significant canonical correlations defines the dimensionality of a phenotypic space in which different subjects are located. In other words, it provides a way of characterising the ‘type’ of each subject along different dimensions. For example, one type of subject may have very precise (hyperprior) beliefs about policy selection and therefore be relatively confident in how they prosecute the visual search. Furthermore, these subjects may adopt a fixed-form (heuristic) search strategy and consequently take a longer time to resolve uncertainty–but will, on average, be more accurate in their decisions. Another type of subject may be more epistemic in nature; reducing their uncertainty about the scene category more efficiently; thereby using shorter scan-path. By simulating responses for characteristic parameter values within the canonical correlation space, one can illustrate the impact of different prior beliefs on behaviour and underlying confidence in decisions and uncertainty about the scene category. In this paper, we focus on normal intersubject variability noting that (in future work) we hope to apply this paradigm to clinical cohorts. Our hope is to show that there are systematic differences in prior beliefs and salience attribution (i.e., the ability to identify salient or epistemically valuable saccadic targets).

### Empirical methods

The experimental design allowed participants to explore the scene by disclosing objects placed at each quadrant of the visual field using eye movements. Upon arriving at a decision, the participants reported their categorisation of the scene using a button box that was placed either to the right or to the left of the head-mount, depending on whether the individuals were right or left-handed.

**Fig 4:**
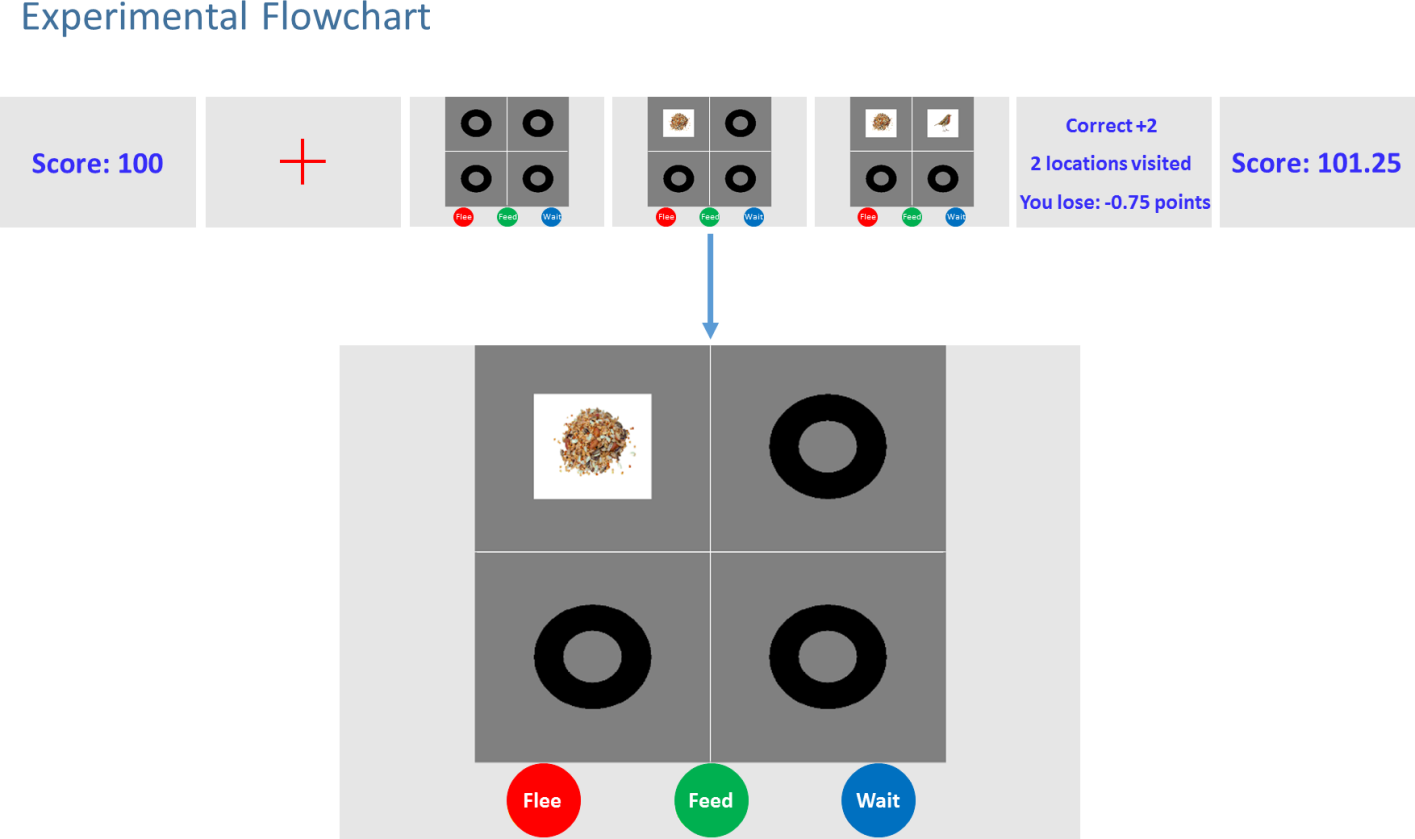
Experiment flowchart. This flowchart shows how a single trial evolves. Firstly, the total score is displayed. Then a fixation cross appears in the centre of the screen. Upon looking at the fixation cross the trial begins. In this particular trial, the participant looks at the top left quadrant and observes a *seed*, and then looks at the top right quadrant and observes a *bird*. At this point it is obvious that the category of the scene is *Feed*. On the fifth action, the participant chooses the *Feed* category by pressing the green button on the controller. This is followed by an auditory feedback, associated with the correct decision. Consequently a feedback screen shows whether the chosen category was correct, the number of quadrants one looked at and the points lost by looking at those quadrants. Finally, the total score is displayed.

Each subject underwent a pre-training phase comprising twenty trials; ensuring that they were accustomed with the experimental setup, head-mount, controller *etc*. They then performed five blocks of the task: two training blocks and then three test blocks. Between each block the subjects rested for a few minutes. Each block consisted of a hundred trials. A fixation cross was displayed on the screen prior to the beginning of a trial. Looking at the fixation cross triggered the trial. After the beginning of the trial, the visual stimuli were displayed in a two-by-two grid; in which each square consisted of a grey dot within a black circle (see Fig 4). The visual display was gaze-contingent; in other words, the grey dots turned into objects (*null*, *bird*, *seed* and *cat*) when looked at. This allowed the subjects to accumulate evidence as they explored the scene within a given trial. At the beginning of a block, the subjects were given 100 points. Subjects were rewarded two points for making a correct categorization and penalized four points for an incorrect one. Both correct and incorrect categorizations were followed by auditory and visual feedback.

We incentivised the participants to sample locations that were more informative using a sampling cost. The penalty of attending to the *n-th* square was given by −0.25 × *n*. The cost of exploration stacked cumulatively as the exploration proceeded; i.e., looking at two squares would cost −0.25 + (−0.5) = −0.75. These task instructions instantiate a particular task set or prior belief that was included in the model or prior preferences (i.e., the probability of not making a decision became less likely with the number of saccades). This introduces a distinction between **C**_**correct**_ (or **C**_**cor**_) and **C**_**quick**_ (or **C**_**q**_) that encode preferences about being *right* or *wrong* and preferences about being undecided as time progresses. See Fig 2.

There was a fixed time limit for each subject of between two and four seconds on each trial. Exceeding this time limit (without choosing) cost the subjects four points. Time limits for each subject were obtained using a staircase procedure during both the training and testing blocks. This staircase procedure was a function of the minimum number of saccades necessary for an efficient categorisation. For instance, if the first object was *bird*, then the most efficient way to explore the scene is to look at the square next to the bird. One only needs to make two saccades to categorize the scene in this case. The number of saccades was summed over 10 trials. Making 10% more saccades than was necessary increased the time limit by 200 ms: it decreased by 200 ms otherwise.

The training and testing blocks differed in two ways. In the training phase, the colours of the buttons on the controller were displayed as dots in the lower half of the screen (below the two-by-two grid scene) with the same colours (and in the same order) as the button press box, to ensure subjects learned to press the correct buttons as quickly as possible. In the testing phase, the coloured dots were removed from the screen and the grid scene was centred on the screen. The sequence of frames in Fig 4 shows the steps of this gaze-contingent paradigm. Stimuli were delivered using Cogent 2000 *(*developed by the Cogent 2000 team at the FIL and the ICN and Cogent Graphics developed by John Romaya at the LON at the Wellcome Department of Imaging Neuroscience) and Psychtoolbox [10,11,12].

#### Subjects

In total 22 subjects were recruited (9 males, 13 females) through the Institute of Cognitive Neuroscience subject database. All subjects gave informed written consent, and the study received ethical approval from the UCL ethics committee (4356/002). The majority of the subjects were students of University College London. The age of the participants ranged between 19 and 57, with mean 25.7 years and standard deviation 9.3 years.

#### Recording method

Subjects were seated 70 cm from the screen on which visual stimuli were displayed–and they rested their chins on a head-mount. Using the Eyelink 1000 eye-tracker their gaze coordinates were recorded as they performed the task. The grid scene was displayed on a 408*mm* × 306*mm* screen with a resolution of 1600x1200. The angle of sight was ≈ 32.5° visual angles horizontally and ≈ 24.4° vertically. The angle of sight of the two-by-two grid scene was ≈ 20.3° during the training phase and ≈ 24.4° during the testing phase, both horizontally and vertically. The size of each object in each square was ≈ 5° and the centre of each object was ≈ 8.5° from the centre in terms of visual angles.

## Results

### Behavioural results

**Fig 5:**
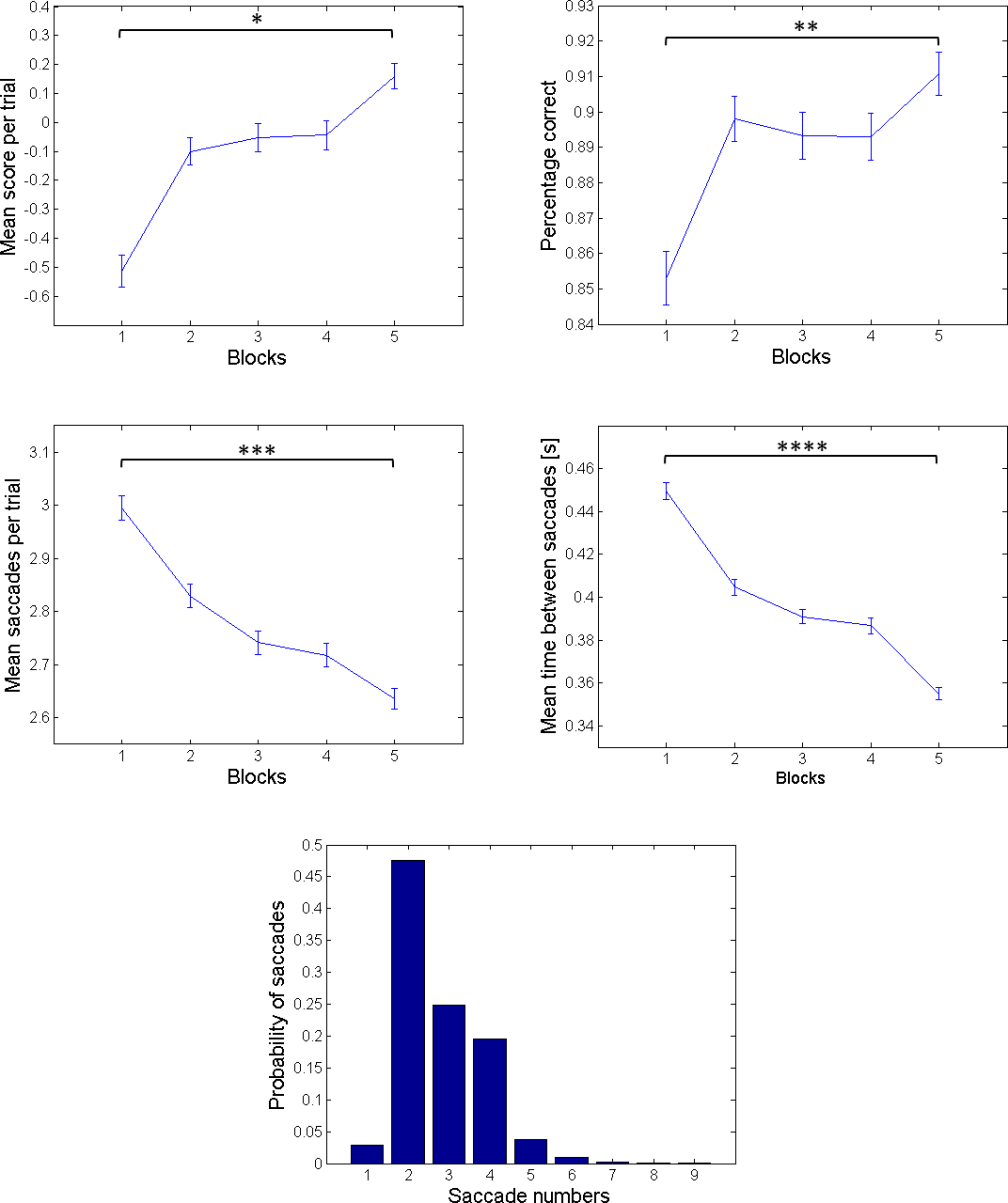
Performance measures. The performance measures in the panels of this figure are averaged over all blocks and subjects. The means and standard error of the means (error bars) are plotted per measure. The top left and right panels show the mean score per trial and the percentage correct categorization over five blocks, respectively (*t*(*df*) = 4388, *p < 0.001; *t*(*df*) = 4393, **p < 0.001). The middle left panel shows the mean saccades per trial (before categorizing the scene) and the middle right panel shows the mean time between saccades in seconds across five blocks (*t*(*df*) = 4392, ***p < 0.001, *t*(*df*) = 7774 ****p < 0.001). The histogram on the bottom panel reports the probability distribution over the number of saccades the participants made before arriving at a decision about the category of the scene.

We first characterised performance across training and testing blocks in terms of their mean score per trial, percentage correct categorizations, mean time interval between saccades and mean number of saccades per trial. These performance measures were averaged over all subjects and are shown across the five blocks in Fig 5. The panels in this figure show that the score per trial increases over blocks and the percentage correct on the fifth block is greater compared to the first block. The middle panels show that both mean saccades per trial and mean time between saccades follow a decreasing trend across blocks. Separate two sample t-tests for these behavioural measures (between the fifth and the first blocks) show that the performance measures in the first and fifth blocks are significantly different. Finally the histogram in the lower panel shows the probability distribution over the saccades.

Given that there are four quadrants in the task and given the subjects do not revisit these quadrants, there can be 4! = 24 different ways of exploring the scene. To account for different types of heuristic strategies, we considered each of these 24 ways of exploring the scene as fixed-form policies. A fixed form (heuristic) policy is specified by taking a fixed action from any given state (e.g., when reading English, one always moves from the current location to the next location on the right). Crucially, this means the probability of state transitions under a fixed form (i.e., heuristic or state action) policy do not change with time. Diverse patterns of exploratory behaviour were observed. Some patterns can be described as heuristics, in that they were used repeatedly within subjects, independently of the context. Other subjects attended to different locations in a seemingly random fashion and some explored in a way to reduce uncertainty about the scene efficiently. Prominent among the heuristics were reading-like and clockwise strategies.

**Fig 6:**
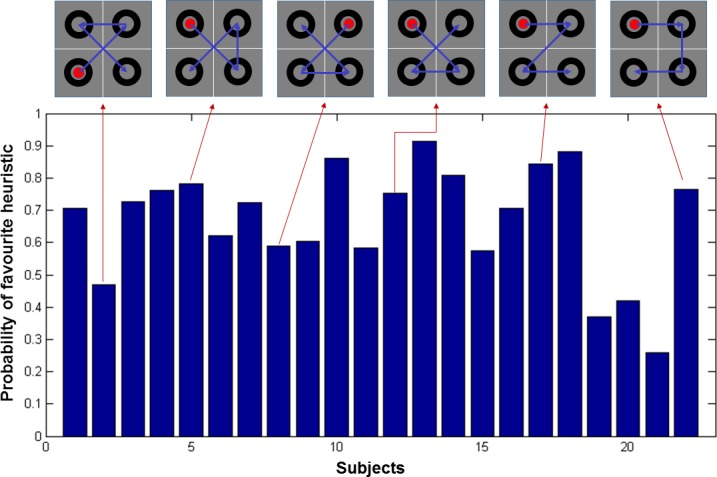
Probability of subjects’ favourite heuristics. The bar plot shows the probability of each participant’s favourite heuristic in terms of the frequency with which the scan-path on a given trial accords with the scan-path of a heuristic strategy. This was repeated over all trials and blocks in each subject. The participants used six distinct heuristic strategies and the scan-paths of these strategies are plotted in the upper panel and linked with (exemplar) subjects that used these strategies.

There are commonalities in the heuristic strategies: e.g. the first two quadrants under the reading and clockwise policies (see the two rightmost scan-paths in Fig 6) are the same. Fig 6 shows the proportion of all trials in which the individual subject’s most commonly used heuristic strategy was employed. There were six distinct heuristic strategies used by 22 subjects. By far the commonest policies are reading-like and clockwise strategies (used 47% and 42% of the time respectively), whereas the next most commonly used heuristic was at 12% (shorter scan-paths can be explained by multiple heuristic policies; hence these percentages do not add up to 100).

### Scan path results

#### Bayesian model comparison, reduction and averaging between block (experience dependent) effects

**Fig 7:**
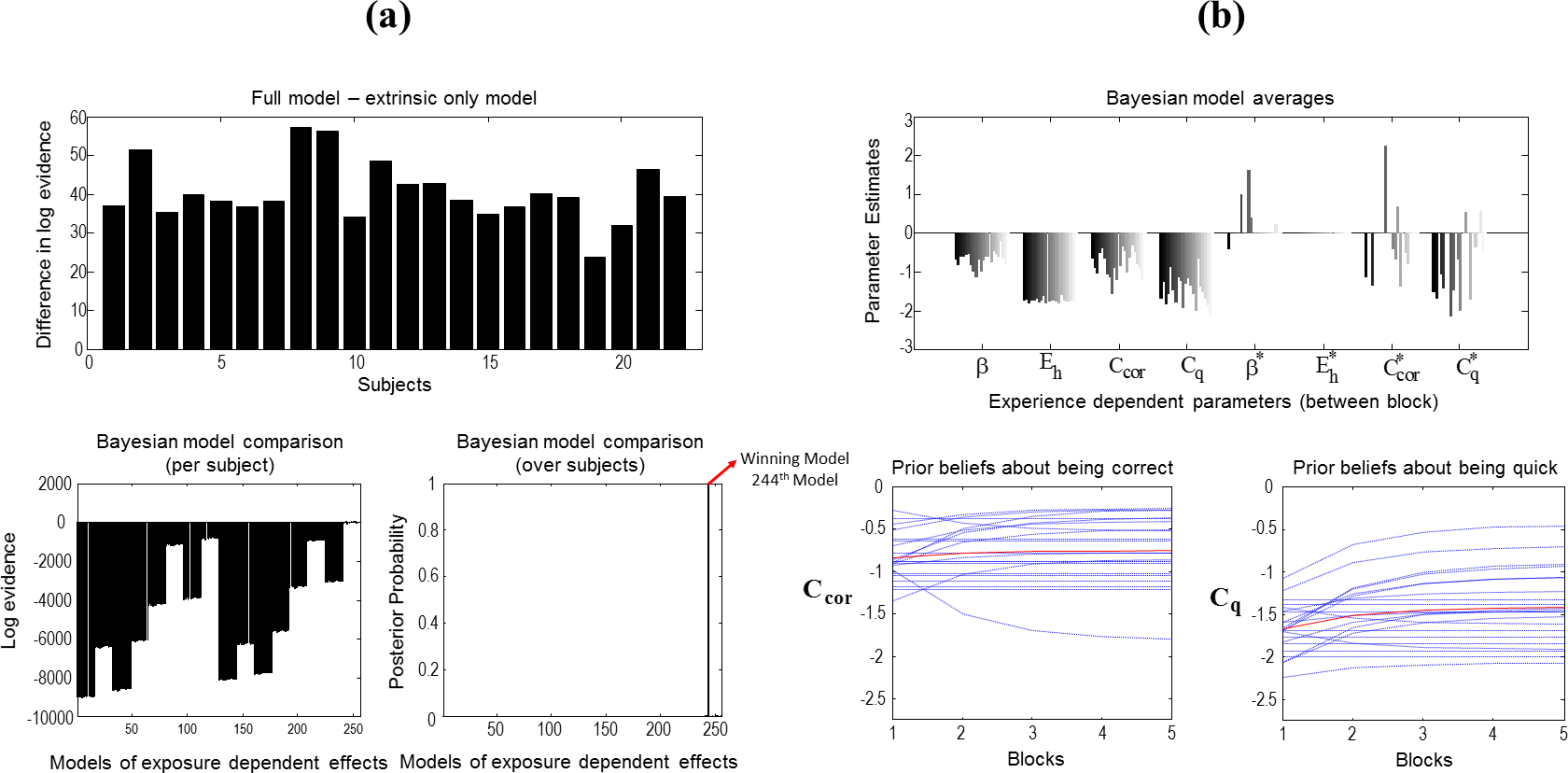
Bayesian model comparison, reduction and averaging. **(a)** The top panel shows the difference between free energies obtained with the full model (epistemic and extrinsic values) and the model with only extrinsic value. The model that contains the epistemic value (full model) has more evidence than the model that does not on an individual basis and over all subjects. Exponential changes in the parameters over blocks where tested using parametric empirical Bayes, implemented with a general linear model that consists of a constant and a decay term. The decay terms are shown with asterisks (e.g. the exposure-dependent changes in the prior precision of beliefs about policies across blocks is denoted by β*, whereas the mean (constant) across blocks is denoted by **β).** This means that there are 4 × 2 = 8 between-block effects. The lower left panel shows the log evidences for all combinations of between-block effects (2^8^ = 256). Here, the model that excludes the change in **β** and **E**_**h**_ (244^th^ model) scores the greatest log evidence. The lower right panel shows the most likely model when a softmax function is applied to the log evidences on the top left panel. **(b)** The upper panel shows the Bayesian model averages for each parameter (in log-space) over subjects after Bayesian model reduction was applied to all 256 models (i.e., redundant parameters, $E_{h}^{*}$ and β* for most participants, were eliminated). Bayesian model reduction automatically eliminates redundant parameters that constitute models with less model evidence. This means that the models that included exposure-dependent changes in the prior precision of beliefs about policies and heuristic bias had a lower log-evidence–and that these parameters were not necessary to explain choice behaviour. The left and right lower panels show the posterior estimates of the scaling coefficients (in log-space) that control the precision of the prior preference matrices about being correct (left) and quick (right).

We first tested whether subjects’ scan paths evidenced the use of epistemic policies. The upper panel of Fig 7A shows the difference between the log evidences obtained with the models that did–and did not–contain epistemic value. The model that incorporates epistemic value had substantially more evidence for every subject. Pooling the log evidence over all subjects the epistemic model scored ≈ 888 more log evidence than the model that contained extrinsic value (i.e., prior preferences) only. This result suggests that the subjects indeed engaged in epistemic visual foraging–and that the epistemic affordance or salience of visual targets was necessary to explain their eye movements.

We then tested whether subjects showed evidence for changes in their prior beliefs from block to block. Fig 7 shows the results of Bayesian model comparison of these between-block or experience-dependent effects. This analysis used parametric empirical Bayes [13] to test for systematic (exponential) changes over blocks prior beliefs; namely, prior inverse precision, heuristic bias and prior preferences–**β**, **E**_**h**_**, C**_**cor**_ and **C**_**q**_. This model of exposure-dependent changes assumes that the greatest change in prior beliefs occurs at the start (in the first blocks) and then plateau in the last blocks. **β** (shown in red in Fig 1A) determines the confidence subjects place in their prior beliefs about policies. **C**_**cor**_ and **C**_**q**_ (shown in red in the lower panel of Fig 2) are scaling coefficients on the log prior preferences about outcomes (in *what* and *where* modalities respectively) that tune the precision of preferences. The higher these parameters, more precise the preferences become. **E**_**h**_ (shown in red in the lower panel of Fig 2) is the final element in the vector of prior preferences over the policies. This encodes the prior propensity for a fixed-form policy (e.g. reading like strategy) specified relative to a value of zero for the remaining (eight) policies.

The changes in these prior beliefs were modelled by specifying a simple general linear model at the between block level that comprised a constant term and a exponential decay with a time constant of one block. The between-block parameters of this hierarchical model comprised a constant and decay parameter for each of the four (prior) parameters at the within-block level. Bayesian model reduction was then used to compare all combinations of the ensuing 4 x 2 = 8 between-block effects, for each subject. There are in total 2^8^ = 256 models.

The results of this analysis are shown in terms of log evidence (pooled or summed over subjects) over the (most likely) 256 models in the lower row, left panel of Fig 7A. These results show that full models (to the right of the bar plot) have much greater evidence than reduced models, with fewer parameters. To assess the most likely model over subjects, we applied a softmax function to the pooled log evidence. The resulting marginal likelihood or model evidence over models and subjects is shown in the lower row, right panel of Fig 7A. This model likelihood suggests that we can be almost certain a nearly complete model is necessary to account for the data. The winning model identified the *changes in* prior precision (**β**) and heuristic bias (**E**_**h**_) over blocks as redundant. This was a little surprising because it suggests that systematic changes in subjects’ preferences–with increasing experience of the paradigm–are expressed largely in terms of their prior preferences (**C**) for being correct or for responding quickly.

The upper panel of Fig 7B shows the Bayesian model averages of the (four) parameters (in log-space) for each subject after Bayesian model reduction was applied to all models. These parameter averages account for uncertainty about the model of between-block effects. The bar plot groups the Bayesian model averages for each parameter over subjects. The first four parameters correspond to the mean or constant effect, while the last four correspond to experience-dependent changes (indicated by asterisks). One can see that there is a remarkably consistent profile of deviations from the prior mean over subjects (first four parameters). However, the experience-dependent effects are less consistent. As would be expected from the Bayesian model comparison, the changes in prior precision and heuristic bias are small; with the Bayesian model averages of heuristic bias (**E**_**h**_) over subjects shrinking to almost zero. The interesting results here are the more consistent and negative parameters controlling the exponential decay of prior preferences (**C**). As the subjects become more familiar or experienced with the paradigm they increase the precision of their prior preferences; especially the prior belief that they will respond more quickly.

These effects are shown in terms of the expected changes in prior preferences over blocks based on subject specific estimates (dotted lines) and the group mean (solid red lines) for prior preferences about being correct (lower left panel) and being quick (lower right panel) in the lower row of Fig 7B. These results suggest that as blocks progress, subjects increase their prior beliefs that they will avoid sampling further (unnecessary) information later in the trials, which can be seen by the increase in **C**_**q**_ over blocks. A key aspect of these subject specific effects is that there is a large intersubject variability that we characterised using canonical correlation analysis.

### Canonical correlation (variates) analysis of between subject effects

**Fig 8:**
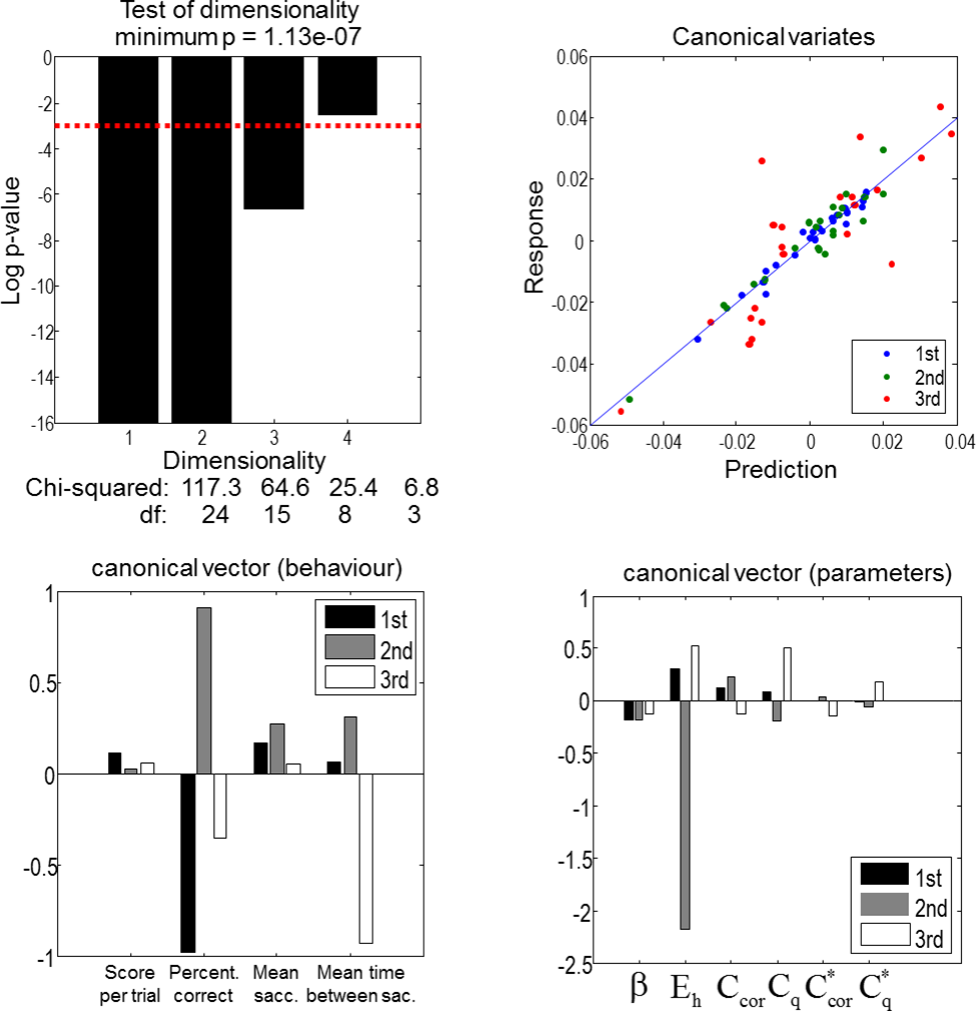
Canonical correlation analysis. The correlations between the Bayesian model average of parameters (among 256 models) and the behavioural measures were analysed using CVA. These parameters are means of the prior inverse precision **β**, heuristic bias **E**_**h**_, scaling coefficients **C**_**cor**_ and **C**_**q**_, and the decay terms $C_{cor}^{*}$ and $C_{q}^{*}$. The behavioural measures are: mean score per trial, percentage correct, mean number of saccades per trial and the mean time interval between saccades. The top left panel shows the results of a chi-squared analysis of the canonical correlations. The first three of four canonical correlations are statistically significant; whereas the fourth canonical correlation is not. The top right panel shows the predicted and observed behavioural canonical variates. The bottom left and bottom right panels show the corresponding canonical vectors. The three canonical variates are each composed of a pair of canonical vectors whose scores on behavioural measure and parameter are illustrated on the left and right bar charts respectively.

Fig 8 illustrates between-subject effects; specifically, the canonical correlations between mixtures of behavioural scores and mixtures of subject specific prior beliefs and experience-dependent changes in those beliefs. This analysis summarised behaviour using four behavioural scores for each subject (normalised to a mean of zero and a sum of squares of one). These scores were as follows:

- Trial performance (mean score per trial).
- Percentage correct (how accurate they were at categorising scenes)
- Mean saccades per trial (number of made saccades before categorizing a scene)
- Mean time between saccades (time period between sampling two consecutive locations in seconds)

These behavioural measures were correlated with the six (normalised) subject-specific Bayesian model averages of the prior beliefs, shown in the lower row right panel of Fig 7A. These estimates correspond to a computational phenotype of each subject.

Canonical correlation analysis (equivalently, canonical variates analysis) established that there were three very significant canonical correlations. In other words, there were three pairs of orthogonal mixtures that could not be accounted for by chance. The significance of these canonical correlations is shown in the upper left panel of Fig 8, in terms of the log probabilities of the four canonical correlations. There are four, because this is the minimum dimensionality of the multivariate variables (i.e., the behavioural measures). It can be seen that the first three canonical correlations are extremely significant. This is reflected in the tight correlations between the predicted and observed behavioural factors (shown on the upper right). The amount of behavioural variance that could be accounted for–in terms of the computational modelling–was incredibly high: 96% for the first canonical pairs of vectors, 92% for the second and 70% for the third. (There is minimal contribution of the score per trial to any of the canonical correlations because this score is explained entirely by two other factors–being correct or not and the number of saccades used.)

The canonical vectors themselves are shown in the lower panels. These correspond to the weights of linear mixtures of the behavioural and computational scores that show the greatest correlation. We have limited these to the three significant correlations (black, grey and white bars). The canonical vectors for the behavioural scores define three behavioural phenotypes (noting that the signs of the canonical vectors are arbitrary):

- The first canonical correlation is driven largely by a correlation between the second behavioural score (percentage correct) and a prior bias towards the heuristic policy. If we flipped the signs of the first (black) canonical vectors, these results suggest that more *accurate* and *quick* subjects are those subjects who, computationally, have a lower prior bias towards heuristic use (**E**_**h**_). In other words, subjects who rely more on epistemic policies tend to categorise scenes more accurately and use fewer saccades.
- The second canonical correlation is dominated by the prior bias towards the heuristic policy that is expressed largely in the percentage correct and, to a certain extent, the mean saccades per trial and time between saccades. This behavioural phenotype is of a *careful* subject who is accurate and takes her time between eye movements. This sort of subject has a large negative heuristic bias (**E**_**h**_); in other words, a careful subject will not appeal to the heuristic search strategies and will prioritise being correct (**C**_**cor**_) over using fewer saccades (**C**_**q**_).
- The third canonical correlation involves the most pronounced experience-dependent changes in beliefs during exposure to the paradigm. This appears to be expressed behaviourally in the percentage correct and time between saccades. This behavioural phenotype is dominated by a negative loading on mean time between saccades and can be regarded as a *hasty* subject, who trades-off between prior beliefs about being correct and being quick in the opposite direction to the careful subject. Crucially, these hasty subjects are the only sort of subjects that change their prior preferences from block to block.

In summary, there is clear evidence that both subjects’ beliefs and their ability to change those prior beliefs with experience have predictive validity in relation to behavioural performance; enabling us to predict most of the behavioural variance between subjects, given their computational phenotyping under the active inference (MDP) scheme.

## Discussion

By fitting models, that did and did not contain epistemic, uncertainty-resolving imperatives for policy selection to saccadic behaviour, we have shown that healthy subjects’ visual exploration–of even simple scenes–provides substantial evidence for the use of epistemic affordance or salience in visual exploration (upper panel of Fig 7A).

Strikingly, a bias towards using heuristic policies to explore visual scenes was associated with lower accuracy (i.e. percentage of correctly categorised trials) in all three canonical variates relating model parameters to behaviour: see the canonical variates in the lower panels of Fig 8. Note that while one might expect heuristic policies would reduce the time between saccades (as in the 2^nd^ and 3^rd^ canonical variates) at the expense of increasing the mean number of saccades (as in the 1^st^ canonical variate), there is no *a priori* reason accuracy should be affected by heuristic use. The association between diminished heuristic use and improved accuracy indicates that having a model of the task structure to direct one’s behaviour not only permits epistemic foraging but also improves overall performance.

We have further shown that one can use canonical correlation analysis to quantify behavioural phenotypes and their underlying computational bases–in this case, the tendency to use efficient epistemic search, the tendency to be careful (using epistemic search but also extra saccades), and the tendency to be hasty but also refine one’s behaviour over blocks.

Finally, we have demonstrated the use of parametric empirical Bayes to infer changes in parameters within subjects over the course of the experiment. Interestingly, subjects did not change their prior beliefs about the inverse precision parameter **β** and the heuristic bias **E**_**h**_ consistently. The change in the subjects’ prior preferences about being ‘correct’ and ‘quick’ best accounts for performance improvements from the first to the last block (lower panel of Fig 7B). In other words, a simple change in the way that people expected themselves to behave was sufficient to explain changes in behaviour. This does not mean to say that the subjects were more confident about their policy selection; rather, they were more confident about the consequences or outcomes of their selected policy.

In recent decades several models have been introduced to explain what may drive visual attention. Some of these models map the features of objects (or image patches) such as colour, intensity, orientation [14,15] motion [16], local contrast and two point intensity correlation [17] onto a saliency map. There are crucial differences between these formulations of salience and ours: First, our model is not designed to process the visual features of the objects–it deterministically knows where it looks (*where*) and what it sees (*what*). Second, unlike early formulations, our model is endowed with a natural curiosity about the hidden causes of the world that drives its visual search and the prior preferences that encourage it to make accurate and timely choices. In short, salience in active inference is an attribute of a policy that has outcomes–not an attribute of stimuli. This is not to say that stimuli do not have an epistemic affordance but it is the sampling of that affordance that is underwritten by salience.

Another perspective on visual attention suggests that cognitive control processes drive visual search behaviour. Under this hypothesis the context in which the visual search tasks are performed drives exploratory behaviour [18–21]. In our paradigm, however, the context is revealed as a *result* of gathering information; in other words, it has to be inferred. Thus a deterministic knowledge of potential contexts, but not the actual context, guides our agent’s search behaviour.

It has also been shown that in a set of expected stimuli, the abrupt appearance and disappearance of an object [22,23] or the presence of improbable stimuli given a context [24] can drive visual attention. These results suggest that novelty or information in Shannon’s terms attract visual attention. However, Itti & Baldi [2] showed that areas of high Bayesian surprise (i.e. that cause greater shifts in beliefs) are more potent attractors of human visual attention; i.e., more salient than informative areas in Shannon’s terms. One difference between the various approaches above is that in Itti and Baldi’s work, Bayesian surprise is computed over low level visual features, rather than hidden states of the world as in active inference. Nevertheless, the principle of Bayesian surprise underwriting visual salience is likely to hold throughout the cortical hierarchy.

This work has some limitations. The small size of the grid scene in our simple visual task limits the potential benefit of using an epistemic strategy–as it is possible to explore all the quadrants in the time allotted. In a larger grid, the contribution of epistemic strategies to exploration may be even more pronounced. For the reasons of simplicity we did not use the explicit distractors (that are uninformative about the scene category)–we simply used a null or grey background. Under the aberrant salience hypothesis of schizophrenia [25], one might predict that subjects with schizophrenia may sample stimuli of no epistemic value: we shall explore this in future work. A more complex visual task may incorporate sub-goals; i.e., utilities attached to objects and not only to *right* and *wrong* feedback. Such tasks may allow more thorough investigation of the exploration/exploitation trade-off. Finally, our visual search model does not explicitly model the processing of visual features of the objects. More ecological paradigms might also incorporate Bayesian surprise about lower level visual features; e.g., uncertainty in the identification of objects themselves.

One may expect that the more a participant engages in heuristic strategies, there would be less behavioural evidence for models that included epistemic value. However, comparing Fig 6 with the upper panel of Fig 7A one does not see such a pattern. There are several reasons why this could be the case. For example, in certain instances, the sequences of fixations are the same under epistemic and heuristic policies. In these situations, it is difficult to disambiguate between epistemic and heuristic policies. More specifically, the simpler explanation for behaviour–afforded by models that included an epistemic component–has greater evidence because it does not require a heuristic bias to explain the observed behaviour. Precluding the heuristic bias affords a more parsimonious explanation (i.e., induces a smaller complexity penalty). Had we used a larger grid, one might have expected greater differences in model evidence among subjects who pursued epistemic policies and those who largely employed heuristic strategies.

In summary, previous work [4], has shown how (synthetic) subjects can evaluate (expected) Bayesian surprise–i.e. epistemic value–and use it to drive Bayes optimal search behaviour or ‘epistemic foraging’. In the current work, we have demonstrated that even in a very simple task, model comparison indicates strong evidence for epistemic foraging (alongside the use of fixed-form or heuristic policies) in healthy subjects. Furthermore, this epistemic foraging is associated not just with more efficient exploration but also with more accurate scene categorisation. In addition, we have shown how canonical correlation analysis can distinguish different behavioural phenotypes and their underlying computational parameters. In the future we hope to use this paradigm in clinical research to investigate aberrant salience attribution in people with a diagnosis of schizophrenia.

This work was part of Innovative Training Network Perception and Action in Complex Environment, supported by the European Union’s Horizon 2020 research and innovation program Marie Sklodowska-Curie Grant Agreement 642961. This paper reflects only the authors’ view and the Research Executive Agency of the European Commission is not responsible for any use that may be made of the information it contains.
